# Thoracic Aortic Disease in Genetic Syndromes Beyond Established Aortopathy Genes

**DOI:** 10.64898/2026.09.04.26362279

**Published:** 2026-09-08

**Authors:** David R. Murdock, Bobbi McGivern, Dongchuan Guo, Kirsty McWalter, Dianna M. Milewicz

## Abstract

**Background:**

Heritable thoracic aortic aneurysms and dissections (HTAD) are caused by rare variants in up to 42 genes, but many affected individuals and families remain genetically unsolved. We sought to determine if aortic phenotypes occurred in cases with *de novo* pathogenic variants in known syndromic genes.

**Methods:**

We queried a clinical exome/genome sequencing database for individuals with *de novo* pathogenic or likely pathogenic variants (PVs) and aortic Human Phenotype Ontology terms. Genes with PVs present in three or more unrelated cases with aortic phenotypes were further evaluated using published evidence, genome-wide rare-variant burden analyses in the UK Biobank and All of Us, and case-level data from independent thoracic aortic disease (TAD) cohorts.

**Results:**

Among 250 individuals with *de novo* PVs and aortic phenotypes, 32 (12.8%) had PVs in genes included on commercial HTAD panels and were excluded from further analyses. The remaining 218 individuals had PVs in 144 genes, including 16 genes identified in three or more unrelated individuals. Genes involved in chromatin regulation and RAS/mitogen-activated protein kinase (MAPK) signaling, as well as several genes associated with overgrowth syndromes, were recurrently observed. Across biobank analyses, independent TAD cohorts, and published reports, the strongest evidence for aortic involvement was observed for *DNMT3A, NF1, PTPN11, PPP1CB, NSD1, ANKRD11, ADNP,* and *ABL1* , including significant rare-variant burden associations for three of these genes. Aortic phenotypes also occurred across multiple Coffin-Siris/BAF-complex genes.

**Conclusions:**

Aortic phenotypes occur across a diverse range of genetic syndromes beyond established HTAD genes. These findings support broader genomic testing in TAD, expansion of HTAD panels to include additional syndromic genes, and consideration of aortic surveillance in select syndromic disorders.

## Full Text Availability

The license terms selected by the author(s) for this preprint version do not permit archiving in PMC. The full text is available from the preprint server.

